# Epidemiological and clinical characterization of a population-based cohort of cutaneous malignant melanoma patients in the South Region of Portugal

**DOI:** 10.1038/s41598-023-32434-6

**Published:** 2023-04-06

**Authors:** Filipa Alves da Costa, Adriana Ramos, Catarina Bernardo, Fábio Cardoso Borges, Ana Costa Miranda, the ROR-Sul network

**Affiliations:** 1 National Cancer Registry and Epidemiology Research Unit, Instituto Português de Oncologia de Lisboa Francisco Gentil, EPE, R. Prof. Lima Basto, Lisboa, Portugal; 2 Research Institute for Medicines (iMED), Faculty of Pharmacy, University of Lisbon, Lisboa, Portugal

**Keywords:** Cancer, Epidemiology

## Abstract

An historical population-based cohort study was conducted aiming to estimate the incidence of cutaneous malignant melanoma in the South Region of Portugal between Jan 2016 and June 2017; to clinically characterize the diagnosed individuals; to describe instituted treatment; and to estimate survival outcomes. Data were extracted from a cancer registry (ROR-Sul) covering 4,800,000 inhabitants (46% of the Portuguese population) and included a total of 789 individuals meeting eligibility criteria. The crude incidence rate (18 months) of melanoma was 13.36/100,000 inhabitants and the Age-Standardized Incidence Rate per 100,000 World population was 9.65/100,000 inhabitants. The most common histological subtypes identified were superficial extension, followed by malignant melanoma and nodular melanoma. Most cases were diagnosed in stage I (50.39%), equally distributed by sex and with a median age of 65 years. During the study period, 174 recurrence events were recorded (23.45%) and recurrence-free survival rate was significantly lower in more advanced stages. Patients had a two-fold risk of recurrence/death when in presence of ulcerated tumors [adjusted hazard ratio (adj HR) = 2.28; 95% confidence interval (CI) 1.40–3.70]. Overall survival rate at 3-years was 80.54% (95% CI 77.58–83.15), higher than previous national reports, and considerably higher for individuals diagnosed at earlier stages (*p* < 0.001). We have also identified differential survival outcomes in stages II–III explained by the uptake of sentinel lymph node biopsy. The epidemiologic and clinical characteristics of malignant melanoma patients studied are consistent with international literature. The incidence and rates observed suggests additional public health campaigns are needed to modify behaviours of the Portuguese population and thus reduce their risk.

## Introduction

Globally, the incidence of melanoma has been increasing^1^. In fact, melanoma formerly known as a rare cancer, it is currently on the top 10 most incident cancers in the US and in many other countries^2^. There is controversy around the influence of sex, where some studies suggest males are at higher risk of developing melanoma^3^. However, most studies indicate that sex differences must be analyzed by age groups and by doing so, there seems to be tendency towards a higher incidence in females until a certain age, which is then inverted^1,4^, possibly explained by cumulative sun exposure. Sex differences may also affect the anatomic site of tumors, as naturally women tend to more often expose lower limbs whereas males tend to expose trunks^5,6^. Various studies have shown that incidence varies also by region. In Europe for example, Nordic countries have the highest incidence and southern ones, including Portugal, Spain and Greece display the lowest values^7^. These differences highlight the importance of detailed epidemiological studies comparing countries and analyzing regions within one country so that meaningful policy measures may be implemented. In fact, it is widely known today that melanoma treatment depends heavily on early diagnosis and on primary prevention so any measures that favor the adoption of responsible exposure behaviors and uptake of screening will likely contribute to improved survival^8^. In the South Region of Portugal, 506 new cases of malignant melanoma were identified in 2010, and 570 in 2011, corresponding to a World-standardized incidence rate of 6.08 and 6.88 per 100,000 inhabitants, respectively, suggesting a possible increase in this period. Survival rates are however, also progressively increasing, with data indicating a 3-year survival rate of 85% in 2010 and 87% in 2011^9^. Of course, various factors may lead to a more reserved prognosis, namely being diagnosed at a later stage, with a more advanced level of invasion (Clark) or a greater tumor thickness (Breslow), and having some specific mutations (e.g. BRAF)^10,11^. Having a deeper knowledge of the clinical characteristics of individuals diagnosed with malignant melanoma will be important to define the necessary strategies for a timely diagnosis and for the institution of the most appropriate treatment to increase the odds of survival. Therefore, this study aimed to estimate the incidence of malignant melanoma in the South Region of Portugal in the period between Jan 2016 and June 2017; to clinically characterize the individuals with this diagnosis; to describe the treatment instituted; and to estimate survival outcomes.

## Materials and methods

### Study design

An historical population-based cohort study was set, using the South-Region Cancer Registry (ROR-Sul) to identify cases diagnosed with malignant melanoma of the skin between 01.01.2016 and 30.06.2017. The expected follow-up period was 3 years, meaning each case was followed since the date of first diagnosis for a period of three years, unless death occurred, or case was lost to follow-up (e.g., left the country).

### Population and sample

The population of interest considered for this study is all individuals residing in the area covered by ROR-Sul.

#### Inclusion criteria

Having a confirmed cutaneous malignant melanoma diagnosis; first diagnosis must have occurred within the time period defined to be of interest for study; included cases must be aged 18 years or older; and live in the area of influence of the ROR-Sul at the moment of diagnosis.

#### Exclusion criteria

Not having histopathological or cytological diagnosis, according to the third revision of the International Classification of Diseases for Oncology^12^.

### Data sources

ROR-Sul is a population-based registry covering 4,800,000 inhabitants (46% of the Portuguese population). Population monitoring is ensured by gathering data originating from 24 hospitals and resorting to data linkage between various sources, namely primary care centers, hospitals (including care provided and diagnostic tests made), and ultimately death certificates. Data linkage can occur by automatic data integration or by manual data entry, in both cases validated by a certified registrar. Whenever a cancer diagnosis is made, regardless of the setting, the case enters the registry and is then prospectively followed throughout the years, contacts made in every point of the health care system are captured, until death occurs. Because cases enter the registry upon diagnosis, previous exposure to risk factors is often not captured.

### Study variables

Variables considered of interest were divided into three main categories. Demographic variables comprised sex, age and district of residence at diagnosis; clinical variables referred to primary tumor location, histological subtype, stage at diagnosis^13^, BRAF mutation, Clark level, Breslow index, ulceration, mitotic index, Lactate Dehydrogenase (LDH) and Eastern Cooperative Oncology Group Performance Status (ECOG PS); treatment variables considered the use of surgery, radiotherapy, other treatment (e.g., electro therapy) or systemic therapy, and combinations of the former. In addition, to these three categories, patient status during the follow-up period was also considered, namely death and disease recurrence.

### Statistical analysis

Demographic, clinical, district distribution and therapeutic characteristics were summarized as medians and interquartile ranges (IQRs) for continuous variables and as absolute and relative frequencies for categorical variables. Crude incidence rates at 18 months were calculated by district of residence and further categorized in three groups, defined according to the amplitude of incidence values estimated, into high [12.35–14.53], medium [10.17–12.35] and low incidence [7.99–10.17], graphically represented using the software GeoDa^14^. Crude incidence rates were computed to consider Age-Standardized Incidence Rate per 100,000 World population (ASRW) for the study region for adults aged over 20 years. The χ^2^ test or Fish exact test was applied to evaluate the association between categorical variables, as applicable, and the non-parametric Wilcoxon test was used for continuous variables. Only variables with missing values below 20% were considered eligible for bivariate analysis, as suggested elsewhere^15^. Median follow-up was computed simply considering time between date of diagnosis and date of death or date of cut-off (3 years after date of diagnosis). Kaplan–Meier estimates have been used to assess recurrence-free survival (RFS) and overall survival (OS). Time of RFS was considered the time elapsed between date of diagnosis and date of recurrence or date of death; disease recurrence was estimated at 3 years for stages I-III, where the event of interest was considered recurrence or metastasis following disease remission. Survival time was considered the time elapsed between date of diagnosis and date of death. For both survival analyses (RFS and OS), patients who do not have the event of interest were censored at last contact date or date of cut-off, as applicable. The log-rank test was used to evaluate RFS and OS differences by stage. Multivariable Cox proportional hazards regression was used to evaluate associations between prognostic variables and RFS separated by stages (I–II and III). The proportional hazard assumptions were verified. All estimated *p*-values were two-sided and a 95% confidence interval (CI) was considered for significance. The software Stata, version 13.0, was used for all statistical analyzes^16^.

### Ethics approval

The current study was conducted according to the principles described in Helsinki Declaration and according to the established national legislation. This implies that informed consent was obtained from all subjects when care delivery is initiated in institutions part of the ROR-Sul network. The study was approved by the institutional review board (IRB) of the Instituto Português de Oncologia de Lisboa Francisco *Gentil* (IPOLFG), on the 2nd of July 2019 (UIC/1232).

## Results

### Population characterization

A total of 789 individuals were included in the analysis. The median follow-up was 36 months and follow-up completeness were 97.5% (n = 770). Cases were equally divided by sex and the median age at diagnosis was 65, similar between males and females.

The most common tumor location was trunk, immediately followed by inferior limbs. Sex was found to influence the tumor location, where most cases located in lower limbs were female (41.50%); in comparison, trunk cases were more frequently identified in males (45.76%) (χ^2^ = 59.16; *p*-value < 0.001). The most common histological subtypes identified were superficial spreading melanoma, followed by malignant melanoma non-otherwise specified and nodular melanoma. The latter was more common among male, whereas acral lentiginous melanoma prevailed among females. In general, females were diagnosed at earlier stages, compared to males. Ulceration was more commonly present among males, among whom higher Breslow indexes were also more frequent (Table 1).

**Table 1:** Demographic and clinical characteristics of cutaneous malignant melanoma cases.

		All cases	Female	Male	*p*-value
Follow-up time, median months (IQR)		36 (36–36)			
Sex, n (%)	Female	400 (50.70)			
	Male	389 (49.30)			
Age, median {Q25–Q75}		65 {52–75}	64	66	0.116
Primary tumor location, n (%)	Trunk	290 (36.76)	112 (28.00)	178 (45.76)	< 0.001*
	Lower extremities	237 (30.04)	166 (41.50)	71 (18.25)	
	Upper extremities	129 (16.35)	69 (17.25)	60 (15.42)	
	Head and neck	119 (15.08)	48 (12.00)	71 (18.25)	
	Other locations	14 (1.77)	5 (1.25)	9 (2.31)	
Histological subtype, n (%)	Superficial spreading	419 (53.11)	218 (54.50)	201 (53.11)	0.052
	Malignant melanoma NOS	149 (18.88)	79 (19.75)	70 (17.99)	
	Nodular melanoma	135 (17.11)	55 (13.75)	80 (20.57)	
	Acral lentiginous melanoma	40 (5.07)	26 (6.50)	14 (3.60)	
	Other subtypes	46 (5.83)	22 (5.50)	24 (6.17)	
Stage at diagnosis, n (%) Unknown, n = 13	I	391 (50.39)	227 (57.61)	164 (42.93)	< 0.001*
	II	216 (27.84)	100 (25.38)	116 (30.37)	
	III	135 (17.40)	56 (14.21)	79 (20.68)	
	IV	34 (4.31)	11 (2.79)	23 (6.02)	
Ulceration, n (%) Unknown, n = 26	Yes	230 (30.14)	102 (26.09)	128 (34.41)	0.012*
	No	533 (69.86)	289 (73.91)	244 (65.59)	
Breslow index, n (%) Unknown, n = 37	≤ 4 mm	585 (77.79)	311 (80.78)	274 (74.66)	0.044*
	> 4.00 mm	167 (21.21)	74 (19.22)	93 (25.34)	
Mitotic index Unknown, n = 234	< 1 mm^2^	280 (50.45)	157 (52.33)	123 (48.24)	
	≥ 1 mm^2^	275 (49.55)	143 (47.67)	132 (51.76)	
Clark index Unknown, n = 273	Level II	135 (26.16)	76 (28.04)	59 (24.08)	–
	Level III	145 (28.10)	84 (31.00)	61 (24.90)	
	Level IV	193 (37.40)	90 (33.21)	103 (42.04)	
	Level V	43 (8.33)	21 (7.75)	22 (8.98)	
BRAF mutations Unknown, n = 600	Wild-type	116 (61.38)	53 (60.92)	63 (61.76)	–
	Non mutated	73 (38.62)	34 (39.08)	39 (38.24)	
LDH Unknown, n = 523	High	245 (92.11)	126 (90.65)	119 (93.70)	–
	Normal	21 (7.89)	13 (9.35)	8 (6.30)	
ECOG PS Unknown, n = 337	0–1	426 (94.25)	212 (95.50)	214 (93.04)	–
	≥ 2	26 (5.75)	10 (4.50)	16 (6.96)	
Recurrences (stages I–III)		115 (15.50)	57 (14.88)	58 (16.16)	0.632
Deaths		152 (19.26)	62 (15.50)	90 (23.14)	**0.007**

Acronyms used in the table: ECOG PS, Eastern Cooperative Oncology Group Performance Status; LDH, Lactate dehydrogenase; NOS Non-Otherwise Specified.

*Signals statistically significant differences. Significant values are in bold.

Among the 189 individuals evaluated for the presence of BRAF mutations, 61.38% (n = 116) were wild type (missing n = 600; 76.05%). Mitotic index was below 1 mm^2^ for 280 cases (50.45%); missing information for this variable was present in 234 cases (29.66%). LDH was normal in most cases (n = 245; 92.11%), even though for 523 cases this data was missing (66.29%). Clark level was also missing for 273 cases (34.60%); for those evaluated level II was identified in 135 cases (26.16%), level III in 145 cases (28.10%), level IV in 193 cases (37.40%) and level V in 43 cases (8.33%). Finally, ECOG PS was the fifth and final variable not possible to consider for bivariate analysis. Missing values were identified in 337 cases (42.71%); in the remaining, the most common was to have PS 0 or 1 (n = 426; 94.25%).

### Incidence rate

**Figure 1:**
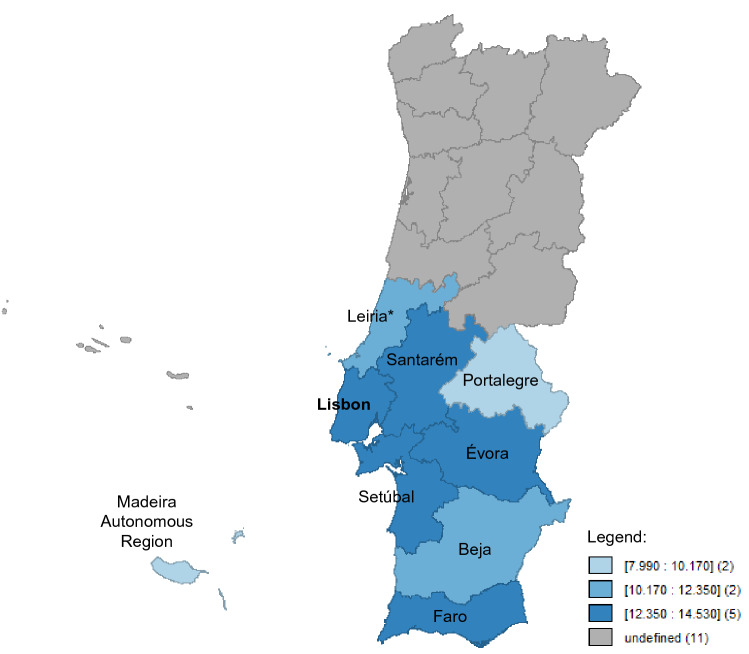
Crude incidence rate of cutaneous melanoma at 18 months in ROR-Sul (figure created by the authors using software GeoDa, release 1.12.1.131, available at https://geodacenter.github.io/).

The crude incidence rate (18 months) of cutaneous melanoma in the South Region was 13.36/100,000 inhabitants and the ASRW was 9.65/100,000 inhabitants. High incidence regions were Lisbon, Évora, Setúbal, Faro and Santarém, with values ranging from 12.40 to 14.53. Low incidence regions were Madeira Autonomous region and Portalegre, with values of 7.99 and 9.34 respectively. The remaining regions (Leiria* and Beja) were classified as medium incidence regions [Fig. 1].

### Treatment patterns

Most patients in this cohort were subject solely to surgery (n = 699; 88.59%). At stages I and II, most patients had only their primary tumour excised (n = 376; 63.62%) or were also subject to the removal of the sentinel lymph node (n = 209; 34.43%). At stages III, most patients were also exclusively treated by surgery (n = 90; 66.67%) and a relevant proportion by surgery and radiotherapy (n = 26; 19.26%). A high proportion of patients at stage IV did not receive any treatment (n = 9; 26.47%), while among the remaining, the most common was to have surgery exclusively (n = 8; 23.53%) or in combination with systemic therapy (n = 7; 20.59%), or other forms of treatment (n = 8; 23.53%) (Table 2).

**Table 2:** Treatment patterns for cutaneous malignant melanoma cases in ROR-Sul.

Treatment patterns	ROR-Sul (n = 789)	Stage I (n = 391)	Stage II (n = 216)	Stage III (n = 135)	Stage IV (n = 34)	Unknown (n = 13)
Surgery only, n (%)	**699 (88.59)**	**379 (96.93)**	**212 (98.15)**	**90 (66.67)**	**8 (23.53)**	**10 (76.92)**
Excising of primary tumour	394 (56.37)	273 (72.03)	103 (48.58)	6 (6.67)	5 (62.50)	7 (70,00)
Excising of primary tumour and lymph node	209 (29.90)	93 (24.54)	100 (47.17)	13 (14.44)	1 (12.50)	2 (20.00)
Excising of primary tumour, lymph node and lymphadenectomy	78 (11.16)	11 (2.90)	5 (2.36)	61 (67.78)	–	1 (10.00)
Other	18 (2.58)	2 (0.53)	4 (1.89)	10 (11.11)	2 (25.00)	–
Surgery and radiotherapy, n (%)	**30 (3.80)**	**1 (0.26)**	**2 (0.93)**	**26 (19.26)**	**1 (2.94)**	**–**
Excising of primary tumour, lymphadenectomy + radiotherapy	13 (43.33)	–	–	13 (50.00)	0 (0.00)	–
Excising of primary tumour, lymph node and lymphadenectomy + radiotherapy	11 (36.67)	–	–	11 (42.31)	–	–
Excising of primary tumour + radiotherapy	5 (16.67)	1 (100)	2 (100)	1 (3.85)	1 (100)	–
Other	1 (3.33)	–	–	1 (3.85)	–	–
Surgery and systemic treatment, n (%)	**21 (2.66)**	–	**1 (0.46)**	**13 (9.63)**	**7 (20.59)**	**–**
Excising of primary tumour, lymph node and lymphadenectomy + Interferon	5 (23.81)	–	–	5 (38.46)	–	–
Excising of primary tumour, lymph node and lymphadenectomy + Clinical trial	3 (14.29)	–	–	3 (23.08)	–	–
Excising of primary tumour + Anti-PD-1/Anti-PD-L1	3 (14.29)	–	–	–	3 (42.86)	–
Other (anti-MEK, anti-BRAF, classical chemotherapy and combinations)	10 (47.62)	–	1 (100)	5 (38.46)	4 (57.14)	–
Other treatment (cryotherapy, laser therapy, nuclear medicine, electrotherapy, and retinoic acid)	**14 (1.77)**	–	**1 (0.46)**	**5 (3.70)**	**8 (23.53)**	–
Without treatment, n (%)	**23 (2.92)**	**11 (2.81)**	–	–	**9 (26.47)**	**3 (23.08)**

Acronyms used in the table: ROR-Sul, South-Region Cancer Registry. Category totals are in bold.

Among the 23 patients that did not receive any treatment (2.92%), the most common reasons were death (n = 6) or patient refusal (n = 3); in 11 patients the reason was unknown.

### Disease recurrence

During the study period, there were 174 recurrence/death events recorded (23.45% of 742). Recurrence-free survival rate was significantly lower in more advanced stages (*p* < 0.001) (Table 3).

**Table 3:** Recurrence of stages I-III cutaneous melanoma at 3 years.

	Total	Stage			*p*-value Logrank test
	n = 742	I	II	III	
		n = 391	n = 216	n = 135	
Events, n (%)	174 (23.45)	24 (6.14)	78 (36.11)	72 (53.33)	
Recurrence-free survival rate at 3 years, % (95% CI)	76.34 (73.10–79.25)	93.72 (90.78–95.75)	63.84 (57.05–69.86)	46.67 (38.08–54.80)	< 0.001

**Figure 2:**
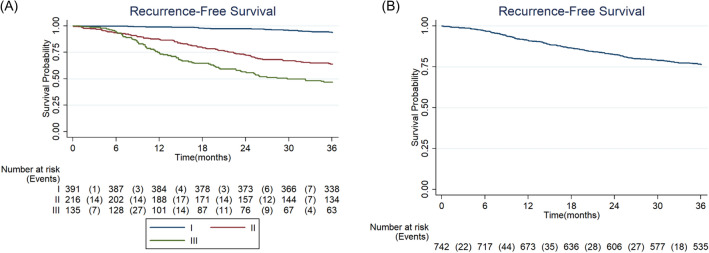
Recurrence-free survival of cutaneous melanoma in ROR-Sul (**A**: overall; **B**: by stage).

Multivariate regression analysis for patients with stages I-II at diagnosis, adjusted for age, demonstrated that presence of ulcerated tumors [adjusted hazard ratio (adj HR) = 3.21; 95% confidence interval (CI) 2.02–5.08] and Breslow index above 4 mm (adj HR = 2.87; 95% CI 1.81–4.54) were associated with a higher risk of recurrence/death. Additionally, multivariate regression analysis for individuals diagnosed at stage III, with the same adjustment variable considered in the previous model, indicated that patients had a two-fold increased risk of recurrence/death when in presence of ulcerated tumors (adj HR = 1.94; 95% CI 1.10–3.44) (Table 4). Figure 2 presents recurrence-free survival by stage.

**Table 4:** Recurrence-Free Survival of cutaneous melanoma by stage (I-II and III)—univariate and multivariate cox regression analysis.

Baseline characteristic	Stage I–II					Stage III				
	Univariate			Multivariate n = 596 number of events = 101		Univariate			Multivariate n = 133 number of events = 71	
	Events	HR (95% CI)	*p*-value	HR adjusted (95% CI)	*p*-value	Events	HR (95% CI)	*p*-value	HR adjusted (95% CI)	*p*-value
Sex										
Female (reference)	102/607	1	0.237	–	–	72/135	1	0.831	–	–
Male		1.26 (0.86–1.86)		–	–		0.95 (0.60–1.52)		–	
Age (years)										
18–40 (reference)	102/607	1	< 0.001*	1		72/135	1	< 0.001	1	
41–65		**1.64** (0.49–5.47)		1.22 (0.36–4.08)	0.745		1.02 (0.43–2.42)		1.04 (0.43–2.46)	0.934
≥ 66		**5.25** (1.66–16.64)		2.66 (0.82–8.58)	0.102		2.22 (1.00–4.93)		1.74 (0.78–3.89)	0.180
Histological subtype										
Nodular melanoma (M8721/3) (reference**)**	102/607	1	< 0.001*	–	–	72/135	1	0.003*	–	–
Superficial spreading (M8743/3)		**0.22** (0.14–0.35)		–	–		**0.46** (0.25–0.83)		–	–
Others		**0.31** (0.19–0.52)		–	–		**1.18** (0.69–2.02)		–	–
Breslow										
0–4 mm ≤ 4 mm (reference)	101/596	1	< 0.001*	1	< 0.001*	71/133	1	0.001*	1	**0.081**
> 4 mm		**6.59** (4.45–9.77)		**2.87** (1.81–4.54)			**2.28** (1.40–3.70)		**1.60** (0.94–2.69)	
Ulceration										
No (reference)	102/607	1	< 0.001*	1		71/133	1	0.001*	1	0.022*
Yes		**5.94** (4.01–8.81)		**3.21** (2.02–5.08)	< 0.001*		**2.52** (1.47–4.31)		**1.94** (1.10–3.44)	

*Statistically significant difference. Acronyms used in the table: HR, Hazard Ratio; CI, confidence interval. Significant values are in bold.

### Survival

**Figure 3:**
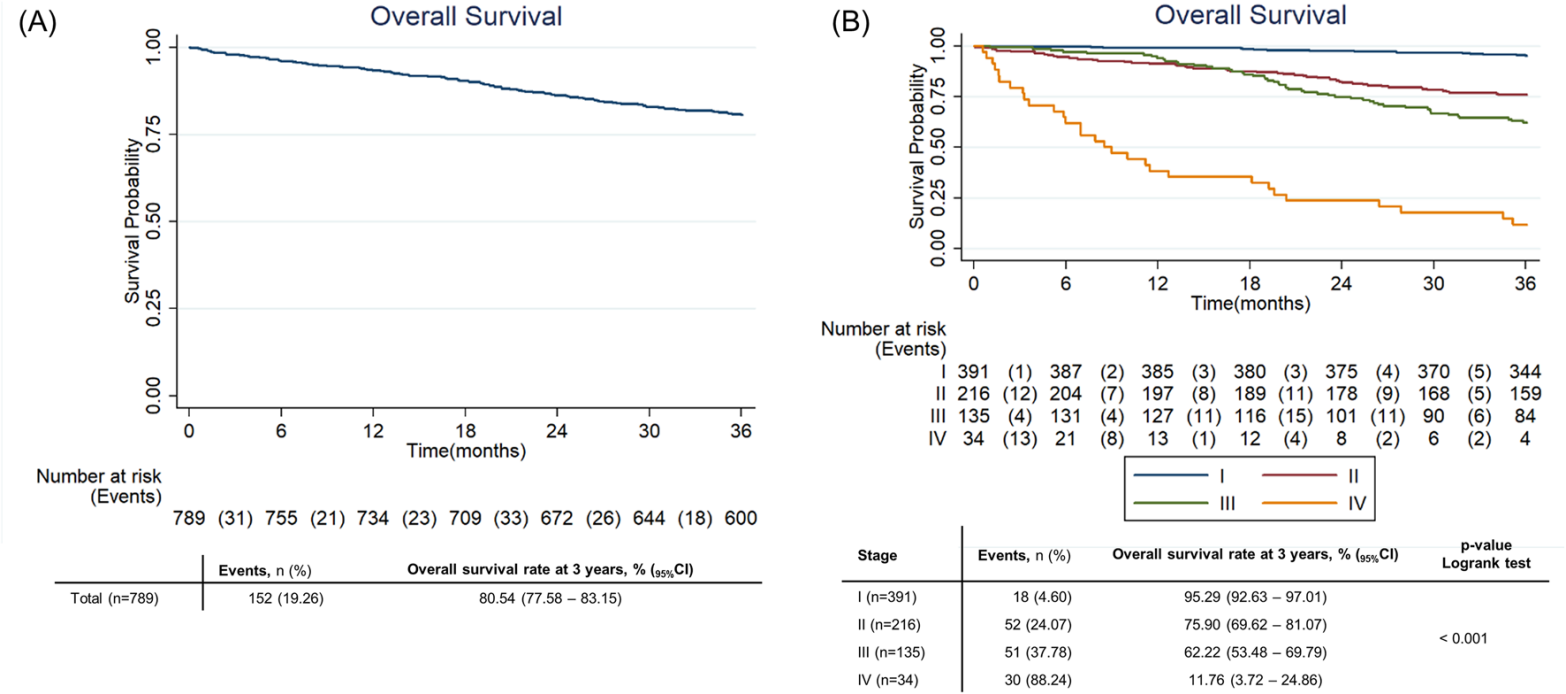
Overall survival of cutaneous melanoma in ROR-Sul (**A**: overall; **B**: by stage).

During the study period, there were 152 death events recorded (19.26%). Overall survival rate at 3-years was 80.54% (95% CI 77.58–83.15). Survival rate was considerably higher for individuals diagnosed at earlier stages (*p* < 0.001), respectively 95.29% (95% CI 92.63–97.01), 75.90% (95% CI 69.62–81.07), 62.22% (95% CI 53.48–69.79) and 11.76% (95% CI 3.72–24.86) for stages 1–IV. Median OS was not reached at 3-years [Fig. 3].

Survival rate was also significantly higher for stage II and stage III cases subject to Sentinel Lymph Node Biopsy (SLNB), respectively 88.07% (CI 80.35–92.89) versus 62.96% (CI 52.22–71.92) and 66.34% (CI 56.23–74.63) versus 50.00% (CI 56.23–74.63) [Supplementary file].

## Discussion

The main findings of this study indicate that in the South Region of Portugal, the median age of diagnosis of cutaneous melanoma is 65 years and that 50% of all cases in the study period were diagnosed at ages between 52 and 75 years of age, in line with previously published data^17,18^. Moreover, it shows that more advanced age significantly increases the odds of recurrence, both at stages I–II and at stage III. The role of age as a prognostic factor has been recognised elsewhere, namely in terms of survival at all stages, and some of the underlying causes seem to be associated with sentinel lymph node positivity (stages I–III) and with responses to treatment (mostly at stage IV)^19^.

In patients with clinical stage I/II melanoma, Sentinel lymph node status is the most significant predictor of survival. Registry data from the Netherlands demonstrates that the uptake of Sentinel Lymph Node Biopsy (SLNB) for these patients has increased by 25% between 2010 and 2016, even though with large regional and age differences^20^. In our study we did not explore regional differences in the uptake of SLNB, but we have explored differences in outcomes. Our data suggests that the uptake of SLNB in stage II is associated with a higher survival rate, confirming previous studies^20^.

The location of the primary tumour identified in this study and its distribution by sex is as expected by previous research and possibly explained by behaviour of sun exposure adopted by men and women^1,2,21,22^. Previous national studies have also reported differences in the location of the primary tumour by sex^23^. Our study has not identified sex as a prognostic factor, contradicting previous research, which has also suggested differential improvements over time, where prognosis of males seem to have improved more due to disparities in instituted treatments^24^. Our sub-analysis of treatment institution was too limited to explore these aspects and should be further studied in the future.

The incidence rate at one year (9.65/100,000 inhabitants) was the same as reported four years earlier for the same region (9.64/100,000 inhabitants)^9^, which seems to contradict previous reports from other countries where an increase in incidence of malignant melanoma has been described^25,26^. Within-country differences in incidence rates could be explained by the different sun exposure patterns and by varied screening practices adopted, primary and secondary prevention, respectively^27^. In fact, the region with the highest incidence identified is the capital, where more intense screening activities have been made available since 2016 between April and October, coinciding with the beach season. As a result, individuals identified in these metropolitan areas were more commonly diagnosed at an earlier stage, hence having a better disease prognosis. This aspect is important to consider also in other countries, considering that policy measures adopted seem to impact on population outcomes.

Notwithstanding, the data also suggests that most cases are diagnosed at early stages, as described in Portugal and also in Sweden^17,23^.

The Oncology Register does not collect information on risk behaviors. The reason for this is that the inclusion of cases in the register is initiated by the diagnosis, thus no previous information is collected. Most of the variables explored in terms of risk factors have ample evidence, namely sun exposure and screening practices and therefore this may not be considered a study limitation.

Treatment patterns observed are aligned with European guidelines published in 2015, recommending surgery in earlier stages (I and II), combined with systemic therapy or radiotherapy in more advanced stages (III) or exclusively for metastatic disease (IV)^28^. However, even though in general terms, the proportion of patients not receiving any treatment was around 3%, patients in stage IV not having access to any treatment was quite high compared to previous registry studies. This discrepancy was even higher for those in this subgroup without access to immune checkpoint blockade and/or BRAF/MEK inhibitors, an aspect that should be further investigated^29^.

Previous studies conducted with German Registry data have suggested that BRAF-mutant patients tend to have better overall survival^30^. Therefore, the fact that only around 25% of patients had BRAF mutation determined may be seen as worrisome. However, in fact, such analysis is only considered crucial for the selection of targeted therapy^31,32^ and this explains why most cases where BRAF mutation was determined were at more advanced stages. Among individuals tested for this mutation, around 40% had a positive result, slightly inferior but still in line with data published for Russia and Denmark^33,34^ and considerably different from reported for Asia^35,36^, which may be a result of the different genomic characteristics of these populations.

To the authors’ best knowledge, there is no national data published on disease recurrence for melanoma, against which our data may be compared. However, the higher recurrence observed at more advanced stages was expected.

This study is innovative and provides valuable data that enables epidemiologic and clinical characterization of malignant melanoma in Portugal. The high coverage of this population-based register, which resorts to data linkage systems, is undoubtedly a strong point of the study, and contributes to the external validity of data presented. It is also worth noting the proportion of follow-up completeness (97.5%), extremely relevant to characterize the outcomes of interest (OS and RFS).

### Limitations

Some of the limitations of this study include its retrospective nature. The low exhaustiveness of some of the variables has been reported elsewhere^37^, and did not allow important prognostic variables, such as LDH and mitotic index, to be included in bivariate and multivariate analysis.

## Conclusion

The epidemiologic and clinical characteristics of malignant melanoma patients included in this study are consistent with the international literature. The observed incidence rate, particularly within country differences, suggests more effective primary and secondary prevention measures, are needed to modify behaviours of the Portuguese population and thus reduce their risk.

## Supplementary Information

Supplementary Information.

## Data Availability

All data included is available in an anonymized manner upon reasonable request sent to the corresponding author.

## Code availability

*GeoDa.* release 1.12.1.131 and StataCorp. *Stata Statistical Software.* College Station, TX: StataCorp LP., 2013. Release 13.
